# Control Work

**Published:** 1897-10

**Authors:** 


### CONTROL WORK.

---

*Pennsylvania.*—A local outbreak of anthrax at Reynoldsville, adjacent to some tanneries, was investigated during September by State Veterinarian Pearson, assisted by Dr. F. F. Hoffman, and placed under control by the adoption of sanitary safeguards.

*Maryland.*—The following communication will explain to the veterinary profession the many newspaper reports current over the country as to the great losses now occurring in Maryland among horses:

DEAR DR. HOSKINS: In reply to your inquiry, the disease in Maryland is the same as you have in Pennsylvania—the same as is written upon by Michener in "Report on Diseases of the Horse," Department of Agriculture. It is much more severe here now, however, than ever before. We are trying hard to find out the cause. The post-mortem results are invariably negative, save a suspicion of inflammation of the stomach. Brain apparently normal. The symptoms are: 1. Drowsiness. 2. Extreme weakness. 3. Loss of power of locomotion; animal down and unable to do more than kick about. 4. Semi-coma. 5. (Generally) coma. 6. Death, in from twenty-four hours to a week; generally about forty-eight hours from first appearance of symptoms. *Etiology:* The appearance of the disease in animals on pasture, and subsiding of the disease when the animals are put in the stable. *Cause:* Poisonous weed. Experiments contemplated in feeding. Will report further observations. Have two veterinarians in the field.

Yours truly,

A. W. CLEMENT.

---

One of our American equine dentists has reached London, and the usual methods of soliciting trade and appealing to owners of horses are being resorted to.

